# Miocene and Pliocene dominated diversification of the lichen-forming fungal genus *Melanohalea* (Parmeliaceae, Ascomycota) and Pleistocene population expansions

**DOI:** 10.1186/1471-2148-12-176

**Published:** 2012-09-11

**Authors:** Steven D Leavitt, Theodore L Esslinger, Pradeep K Divakar, H Thorsten Lumbsch

**Affiliations:** 1 The Field Museum, Department of Botany, 1400 S. Lake Shore Drive, Chicago, IL, 60605, USA; 2 Department of Biological Sciences #2715, North Dakota State University, PO Box 6050, Stevens Hall, Fargo, ND, 58108-6050, USA; 3 Departamento de Biología Vegetal II, Facultad de Farmacia, Universidad Complutense de Madrid, Madrid, 28040, Spain

**Keywords:** Bayesian skyline plot, BEAST, Divergence times, Coalescence, Concatenation, Lichens, *Melanohalea*, Pleistocene, Reproductive strategy, Species tree

## Abstract

**Background:**

Factors promoting diversification in lichen symbioses remain largely unexplored. While Pleistocene events have been important for driving diversification and affecting distributions in many groups, recent estimates suggest that major radiations within some genera in the largest clade of macrolichens (Parmeliaceae, Ascomycota) vastly predate the Pleistocene. To better understand the temporal placement and sequence of diversification events in lichens, we estimated divergence times in a common lichen-forming fungal genus, *Melanohalea*, in the Northern Hemisphere. Divergence times were estimated using both concatenated gene tree and coalescent-based multilocus species tree approaches to assess the temporal context of major radiation events within *Melanohalea*. In order to complement our understanding of processes impacting genetic differentiation, we also evaluated the effects of Pleistocene glacial cycles on population demographics of distinct *Melanohalea* lineages, differing in reproductive strategies.

**Results:**

We found that divergence estimates, from both concatenated gene tree and coalescent-based multilocus species tree approaches, suggest that diversification within *Melanohalea* occurred predominantly during the Miocene and Pliocene, although estimated of divergence times differed by up to 8.3 million years between the two methods. These results indicate that, in some cases, taxonomically diagnostic characters may be maintained among divergent lineages for millions of years. In other cases, similar phenotypic characters among non-sister taxa, including reproductive strategies, suggest the potential for convergent evolution due to similar selective pressures among distinct lineages. Our analyses provide evidence of population expansions predating the last glacial maximum in the sampled lineages. These results suggest that Pleistocene glaciations were not inherently unfavorable or restrictive for some *Melanohalea* species, albeit with apparently different demographic histories between sexually and vegetatively reproducing lineages.

**Conclusions:**

Our results contribute to the understanding of how major changes during the Miocene and Pliocene have been important in promoting diversification within common lichen-forming fungi in the northern Hemisphere. Additionally, we provide evidence that glacial oscillations have influenced current population structure of broadly distributed lichenized fungal species throughout the Holarctic.

## Background

Lichenized fungi form mutualistic relationships with photoautotrophic organisms (photobionts), mainly green algae (Trebouxiophyceae and Ulvophyceae) and/or cyanobacteria. The lichen symbiosis has been highly successful within fungi, especially Ascomycota, with more than 18,000 currently accepted species [1] and an estimated diversity of greater than 28,000 species [2]. Due to the availability of genetic data and analytical improvements, DNA-based approaches play an increasing role in the recognition of diversity in lichenized fungi that would otherwise be impossible to recognize using classical phenotypic characters due to morphological convergence or parallelism [3-11].

In spite of the recent advancements in recognizing diversity in lichen-forming fungi, assessing diversification within a temporal context remains largely unexplored in nearly all groups of these important fungal symbionts, (exceptions include [12-14]). This is largely due to the poor fossil record for lichenized fungi, and also fungi in general, and uncertainties in the interpretation of the few known fossil records [15-17]. However, the timing of speciation events plays a valuable role, complementary to discovering and describing diversity, by addressing biogeographical, climatic, ecological, and other factors associated with diversification and extinction within a temporal context, (e.g. [12,18-20]).

In spite of difficulties in obtaining accurate estimates of divergence times [21,22], recent analytical advances in using relaxed molecular clocks, the inclusion of multiple fossil calibrations, and informative priors on substitution rates have improved accuracy in molecular dating [23-26]. However, a recent study also suggested that methods that fail to incorporate the process of gene lineage coalescence may provide inaccurate estimates of divergence dates [18]. While gene trees, including phylogenies estimated from concatenated sequence data, can overestimate divergence times because they do not correct for genetic divergence that predates speciation, species tree methods incorporating the process of gene lineage coalescence likely provide a more biologically realistic framework for dating divergence events [18].

Within lichen-forming ascomycetes, Parmeliaceae (Lecanorales) constitutes one of the largest and best-studied families [27-32]. Although some lichen-forming fungal lineages have geographically restricted distributions, (e.g. [10,14]), there is mounting evidence that transoceanic dispersal has commonly occurred within some lichenized fungi and has played a major role in diversification within Parmeliaceae [12,33,34].

Unrecognized diversity is common in many phenotype-based species circumscriptions in the family, confounding the current interpretation of ecological and biogeographical patterns [7,9,10,35,36]. Additionally, interpreting biogeographical patterns and factors driving diversification is further complicated by a common pattern of long-distance dispersal in many taxa in Parmeliaceae, (e.g. [12,33,34,37]). Therefore, accurate estimates of divergence times are especially critical for identifying major factors driving diversification. A recent study investigating the origin and diversification in the largest clade of macrolichens, the parmelioid lichens (Parmeliaceae, Ascomycota), provides valuable taxon-specific estimates of substitution rates for three genetic markers based on three calibration points, including two dated fossils from Parmeliaceae [12].

Within Parmeliaceae, the genus *Melanohalea* O. Blanco et al. includes 22 species based on traditional taxonomy, most of which occur primarily on bark and wood throughout the Holarctic [38-44]. Only four known taxa occur in the southern Hemisphere [45-47]. Many *Melanohalea* species display broad geographic and ecological distributions, although a limited number of taxa appear to have more restricted ranges [43]. Otte et al. [43] suggested that distribution patterns in *Melanohalea* are largely determined by contemporary ecogeographical factors, and most species have largely filled their potential distributions in the northern Hemisphere. Furthermore, the distributions of some *Melanohalea* taxa, including *M. elegantula* (Zahlbr.) O. Blanco et al. and *M. exasperatula* (Nyl.) O. Blanco et al., have been linked to eutrophication, air pollution, and other anthropogenic factors [48-52].

While Pleistocene events have been shown to have been important factors driving diversification and affecting distributions in many groups, (e.g. [18,53-55]), recent estimates suggest that the *Melanohalea* radiation vastly predates Pleistocene glacial cycles [12]. Within many parmelioid genera, major radiations occurred from the Late Oligocene to the early Pliocene, before the climate became cooler, drier, and more seasonal at the end of the Pliocene [12,56]. However, the overall importance of Pleistocene glacial cycles in diversification within *Melanohalea* is unknown.

Reproductive strategies vary among *Melanohalea* taxa. Sexual reproduction is restricted to characteristic fungal fruiting bodies (ascomata) producing meiospores (=ascospores), and is common in at least 13 of the 22 described species. Ascospores are dispersed independent of the photosynthesizing partner (photobiont) and require reacquisition of the appropriate photobiont partner in order to re-establish the lichenized condition. In contrast, other species within *Melanohalea* commonly propagate asexually by means of vegetative diaspores, either isidia or soredia. These specialized vegetative reproductive propagules contain both fungal and algal symbionts, eliminating the need for independent acquisition of the appropriate photobiont partner. The isidiate taxa *M. elegantula* and *M. exasperatula* show a remarkable potential for dispersal and may be spreading in some areas [43,48,49]. Poelt [57] hypothesized that lichenized fungi reproducing asexually (via soredia and isidia) are generally more successful in pioneering formerly glaciated areas than forms that reproduce sexually. In contrast, Nimis [58] argued that the distribution of Holarctic lichens is more likely determined by general ecology than by their reproductive strategy alone. The latter argument supports the assumption that the distribution of *Melanohalea* within the Holarctic today widely reflects their biological constitution, rather than their geographic origin or dispersal strategy [43]. However, population structure and history is poorly understood in most lichen-forming ascomycetes and the role of their reproductive strategy in response to climate fluctuation remains unclear.

Cryptic lineages within phenotypically circumscribed taxa are common in Parmeliaceae [35], and previously unrecognized species-level lineages have now been recognized within six of the phenotype-based *Melanohalea* species from a broad sampling of populations in the northern Hemisphere [59]. Diversity within this genus is now well-characterized, at least in the northern Hemisphere, providing an excellent study system with which to test the relative influence of Miocene orogeny and climatic conditions and Pleistocene glacial cycles on common lichen-forming fungi.

In this study our goals are twofold: (1) we aim to estimate divergence times in the lichenized genus *Melanohalea* using both concatenated gene tree and coalescent-based multilocus species tree approaches; and (2) we evaluate the impact of Pleistocene glacial cycles on the population demography among four common sexually reproducing lichen-forming fungal species and two taxa reproducing largely via vegetative diaspores. With estimates of divergence times, we examine the relative roles of Miocene orogeny and climate change and Pleistocene glacial cycles on diversification in the lichenized fungal genus *Melanohalea*. We are also interested in population demographic changes in common *Melanohalea* species after the last glacial maximum (LGM), including anthropogenic factors. Here we present estimates of divergence times within *Melanohalea* and assess population demographic histories in relation to the LGM.

## Results

The complete ITS data matrix consisted of 487 sequences and 511 aligned nucleotide position characters (Additional file 1; TreeBase ID: 12364). The six locus data matrix, representing genetic diversity identified from the ITS gene tree, consisted of 138 samples and 3839 aligned nucleotide position characters (Additional file 2; TreeBase ID: 12364). All sequences generated for this study have been deposited in GenBank under accession Nos. JQ812998 – JQ814066. Table 1 summarizes patterns of variation in sampled loci and the best-fit model of evolution selected using the Akaike Information Criterion (AIC) in jModeltest.

**Table 1:** **Genetic variability in Melanohalea of sampled markers used in this study, including: the number of specimens, *N *, alignment length (number of base pairs); variable and parsimony-informative (PI) sites for each sampled locus; and locus-specific model of evolution identified using the Akaike information criterion in jModeltest**

**Locus**	***N*(# of unique haplotypes)**	**Aligned length**	**# of variable sites**	**# of PI sites**	**Model selected**
ITS (Total)	487 (176)	511	191	153	TIM1ef + I + G
ITS	137 (99)	511	179	146	TIM1ef + I + G
nuLSU	126 (57)	529	65	52	TPM2 + G
mtSSU	101 (33)	794	68	55	TPM1uf + I
*MCM*7	105 (72)	514	151	132	TIM3ef + I
*RPB*1	109 (59)	775	223	186	TIM2ef + I + G
*RPB*2	103 (53)	716	182	155	TPM2uf + I + G

### Age estimates on concatenated and species topologies

High posterior effective sample sizes (ESS > 200) were observed for all parameters in the BEAST analyses. The substitution rates in *Melanohalea* of the six sampled loci (ITS, nuLSU, mtSSU, *MCM*7, *RPB*1, and *RPB*2), estimated under a relaxed clock, are shown in Table 2. The estimated substitution rate in the nuclear ITS was nearly five times higher than the mtSSU and nuLSU, and over two times higher than the sampled protein-coding loci. The protein-coding markers had relatively similar estimated substitution rates.

**Table 2:** **Estimates of substitution rates in *Melanohalea *from the BEAST analysis of the concatenated six-loci dataset estimated under a relax molecular clock using fixed substitution rates for the nuLSU, mtSSU, and *RPB *1 markers **[12]

	**Concatenated gene tree analysis**		**Multilocus species tree analysis**	
Locus	Rate	Rate 95% HPD	Rate	Rate 95% HPD
ITS	3.296	2.49 – 4.11	3.41	2.67 – 4.17
LSU	0.684	0.58 – 0.80	0.71	0.62 – 0.80
mtSSU	0.701	0.42 – 1.05	0.66	0.44 – 0.92
*MCM*7	1.649	1.26 – 2.05	1.71	1.36 – 2.08
*RPB*1	1.548	1.27 – 1.82	1.70	1.57 – 1.83
*RPB*2	1.391	1.08 – 1.75	1.51	1.20 – 1.82

Units: substitution/site/year X 10^-9^. HPD: highest posterior density interval.

**Figure 1:**
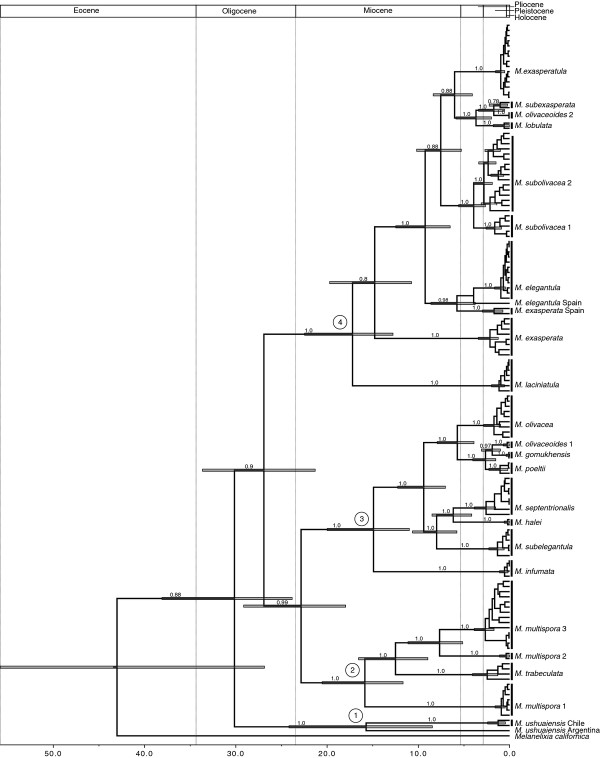
**Dated BEAST maximum clade credibility tree for *Melanohalea *estimated from concatenated data. ** The chronogram was estimated from a partitioned data set consisting of six loci (ITS, nuLSU, mtSSU, *MCM*7, *RPB*1, *RPB*2) under a relaxed molecular clock. The divergence times correspond to the mean posterior estimate of their age in millions of years. The bars indicate the 95% HPD interval for the divergence times estimates. The four major *Melanohalea* clades identified in this study are indicated by ‘1’, ‘2’, ‘3’, and ‘4’ at the corresponding node. Values on branches indicate posterior probability, and only support indices posterior probability values > 0.50 are presented.

**Figure 2:**
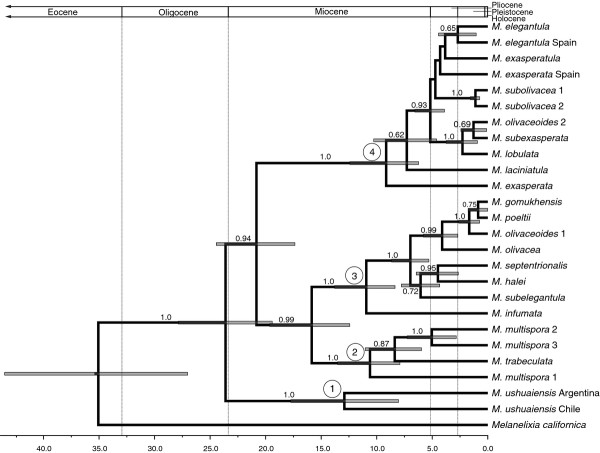
**Dated multilocus coalescent-based species tree for *Melanohalea. *** The chronogram was estimated from a multilocus data (ITS, nuLSU, mtSSU, *MCM*7, *RPB*1, *RPB*2) within a coalescent-based framework in *BEAST. The divergence times correspond to the mean posterior estimate of their age in millions of years. The bars indicate the 95% HPD interval for the divergence times estimates. The four major *Melanohalea* clades identified in this study are indicated by ‘1’, ‘2’, ‘3’, and ‘4’ at the corresponding node. Values on branches indicate posterior probability, and only support indices posterior probability values > 0.50 are presented.

Our calibrated maximum clade credibility chronograms from analyses of the concatenated data matrix (ITS, nuLSU, mtSSU, *MCM*7, *RPB*1, *RPB*2) and multi-locus species-tree approach are shown in Figures 1 and 2, respectively. Divergence times estimated from the species tree approach were between 0.2 and 8.3 million years more recent across the genus, with smaller differences at more recent timescales, i.e. divergences that occurred during the Pleistocene (Figures 1 and 2; Table 3).

**Table 3:** **The dates of origin of lineages of *Melanohalea *from their initial divergence (ancestral splits; stem origin) and the time to the most recent common ancestor (tmrca) of extant haplotypes**

**Lineage**	**Stem origin (MYA) concatenated**	**Stem origin (MYA) coalescent**	**tmrca extant haplotype (MYA) concatenated**
*Melanelixia* – *Melanohalea* split	43.5 (95% HPD = 26.9-59.7)	35.2 (95% HPD = 27.0-43.5)	NA
Origin of *Melanohalea*	30.2 (95% HPD = 23.8- 38.1)	**23.7 (95% HPD = 19.4-27.9)**	NA
*M. elegantula*	3.90 (−)	2.7 (95% HPD = 1.0-4.4)	**1.0 (95% HPD = 0.5-1.6)**
*M. elegantula* Spain	3.9 (−)	2.7 (95% HPD = 1.0-4.4)	-
*M. exasperata*	14.8 (95% HPD = 10.8-19.7)	**9.2 (95% HPD = 6.2-12.4)**	**2.2 (95% HPD = 1.2-3.4)**
*M. exasperata* Spain	**5.8 (95% HPD = 3.8-8.6)**	4.2 (−)	**1.7 (95% HPD = 0.7-3.0**
*M. exasperatula*	6.0 (95% HPD = 4.1-8.4)	3.7 (−)	**1.0 (95% HPD = 0.5-1.5)**
*M. gomukhensis*	**1.9 (95% HPD = 1.0-3.1)**	0.9 (95% HPD = 0.0-1.8)	**0.1 (95% HPD = 0.0-0.4)**
*M. halei*	**6.2 (95% HPD = 4.2-8.5)**	**4.5 (95% HPD = 2.7-6.4)**	**0.2 (95% HPD = 0.0-0.3)**
*M. infumata*	**14.9 (95% HPD = 11.0-20.0)**	**11.0 (95% HPD = 8.4-13.8)**	**0.6 (95% HPD = 0.2-1.1)**
*M. laciniatula*	**17.2 (95% HPD = 12.8-22.5)**	7.4 (95% HPD = 4.6-10.3)	**1.2 (95% HPD = 0.6-2.0)**
*M. lobulata*	**3.7 (95% HPD = 2.0-5.9)**	**2.3 (95% HPD = 0.9-3.7)**	**0.6 (95% HPD = 0.0-1.8)**
*M. multispora* 1	**15.9 (95% HPD = 11.7-20.5)**	**10.6 (95% HPD = 7.9-13.5)**	**0.9 (95% HPD = 0.5-1.6)**
*M. multispora* 2	**7.7 (95% HPD = 5.1-11.1)**	**5.1 (95% HPD = 2.8-7.3)**	**0.4 (95% HPD = 0.0-1.1)**
*M. multispora* 3	**7.7 (95% HPD = 5.1-11.1)**	**5.1 (95% HPD = 2.8-7.3)**	**2.7 (95% HPD = 1.7-3.9)**
*M. olivacea*	**5.7 (95% HPD = 3.9-7.9)**	**4.2 (95% HPD = 2.7-5.8)**	**1.7 (95% HPD = 1.0-2.8)**
*M. olivaceoides* 1	**1.9 (95% HPD = 1.0-3.1)**	**1.7 (95% HPD = 0.7-2.6)**	**0.3 (95% HPD = 0.0-0.7)**
*M. olivaceoides* 2	**1.7 (95% HPD = 0.6-3.4)**	1.3 (95% HPD = 0.1-2.4)	**0.1 (95% HPD = 0.0-0.3)**
*M. poeltii*	**2.6 (95% HPD = 1.5-4.0)**	0.9 (95% HPD = 0.0-1.8)	**1.0 (95% HPD = 0.2-2.3)**
*M. septentrionalis*	**6.2 (95% HPD = 4.2-8.5)**	**4.5 (95% HPD = 2.7-6.4)**	**2.6 (95% HPD = 1.5-3.9)**
*M. subelegantula*	**8.0 (95% HPD = 5.5-10.6)**	6.0 (95% HPD = 4.3-7.8)	**1.3 (95% HPD = 0.6-2.3)**
*M. subexasperata*	**1.7 (95% HPD = 0.6-3.4)**	1.3 (95% HPD = 0.1-2.4)	1.0 (95% HPD = 0.2-2.2)
*M. subolivacea* 1	**3.9 (95% HPD = 2.7-5.6)**	**1.1 (95% HPD = 0.7-1.6)**	**1.6 (95% HPD = 0.9-2.6)**
*M. subolivacea* 2	**3.9 (95% HPD = 2.7-5.6)**	**1.1 (95% HPD = 0.7-1.6)**	**2.4 (95% HPD = 1.9-4.0)**
*M. trabeculata*	**12.5 (95% HPD = 9.0-16.6)**	8.4 (95% HPD = 6.0-11.0)	**2.5 (95% HPD = 1.3-4.1)**
*M. ushuaiensis* Argentina	**15.7 (95% HPD = 8.5-24.2)**	**12.9 (95% HPD = 8.0-17.7)**	-
*M. ushuaiensis* Chile	**15.7 (95% HPD = 8.5-24.2)**	**12.9 (95% HPD = 8.0-17.7)**	**1.3 (95% HPD = 0.5-2.4)**
Split 1	**15.7 (95% HPD = 8.5-24.2)**	**12.9 (95% HPD = 8.0-17.7)**	NA
Split 2	**15.9 (95% HPD = 11.7-20.5)**	**10.6 (95% HPD = 7.9-12.4)**	NA
Split 3	**14.9 (95% HPD = 11.0-20.0)**	**10.9 (95% HPD = 8.4-13.8)**	NA
Split 4	**17.2 (95% HPD = 12.8-22.5)**	**9.1 (95% HPD = 6.2-12.4)**	NA

**Bolded** values correspond to posterioir support values greater than 95%;.

‘-’ corresponds to posterioir support less than 50%.

The divergence of *MelanelixiaMelanohalea*, estimated from the concatenated six gene dataset, occurred ca. 43.5 (95% HPD = 26.9-59.7) million years ago (Ma) and is similar to a previous estimate of 41.55 Ma [12]. However, in the multilocus species tree approach this split was estimated to have occurred more than eight million years more recently, ca. 35.2 (95% HPD = 27.0-43.5) Ma (Table 3). Divergence dates estimated in the concatenated multilocus analysis support the initial radiation of *Melanohalea* during the Oligocene, ca. 30.5 (95% HPD = 23.8-38.1) Ma, while divergence estimates from the multilocus species tree approach suggests a more recent radiation during the Oligocene-Miocene boundary, ca. 23.7 (95% HPD = 19.4-27.9) Ma (Table 3). Both the concatenated and species tree approaches suggest that most diversification of *Melanohalea* occurred throughout the Miocene and Pliocene, and divergence estimates suggest that diversification during the Pleistocene glacial cycles was not accompanied by speciation in *Melanohalea* (Figures 1 and 2). Divergence dates from the multilocus concatenated analysis suggests a relatively synchronous initial radiation of the four major clades in *Melanohalea* with mean diversification times occurring between 14.9 – 17.2 Ma (Figure 1), with no clear temporal pattern in subsequent diversification events. However, dates estimated from the species tree approach provide a somewhat different perspective of diversification, with mean diversification times occurring between 9.1 -12.9 Ma (Table 3). The greatest disparity found in estimates was in the radiation of clade IV, estimated to begin radiating 17.2 (95% HPD = 12.8-22.5) and 9.1 (95% HPD = 6.2-12.4) in the concatenated and species trees approaches, respectively (Figure 2).

In the dated chronogram estimated from concatenated data, species divergences that occurred during Pleistocene glacial cycles were limited to two distinct clades, a clade containing *M. subexasperata* and *M. olivaceoides *2 and a clade with *M. gomukhensis*, M*. olivaceoides *1, and *M. poeltii*. Dates estimated in the species tree analysis suggest Pleistocene divergence for two additional clades, a split between the two lineages in the *M. subolivacea* complex and a split between *M. elegantula* and *M. elegantula *Spain (Table 3).

### Historical demography

Genetic diversity indices (*H*d, *S*, and *π*) for lineages previously recognized in [59] are summarized in Table 4. Overall, lineages producing sexual fruiting structures (ascomata) showed greater nucleotide and haplotype diversities, relative to lineages that commonly reproduce via vegetative diaspores (isidia/soredia). However, haplotype diversity was relatively high in the isidiate taxon *M. laciniatula* (0.833), and, in contrast, haplotype diversity was low in the apotheciate lineage *M. multispora *1 (0.143).

**Table 4:** **Estimates of genetic diversity for sampled lineages within *Melanohalea ***

**Taxon**	***N***	***H***	***Hd***	***S***	***π***	**Tajima's*D***	**Fu and Li's*Fs***	***Fs***	**RI**	**SSD**
*Melanohalea* (total)	487	146	0.956	142	0.0481	-	-	-	-	-
M. elegantula*	100	16	0.478	15	0.0017	**−2.1366**	**−3.0108**	**−17.149**	0.09731746	**1.82E-05**
*M. elegantula* Spain	1	NA	NA	NA	NA	NA	NA	NA	NA	NA
*M. exasperata*	11	8	0.927	21	0.0140	−0.9720	−1.3005	−1.144	0.11438	0.031023
*M. exasperata* Spain	2	2	1	9	0.0224	NA	NA	NA	NA	NA
M. exasperatula*	105	13	0.756	17	0.0039	−1.5449	−1.9654	−4.29	0.068649	**0.012024**
*M. gomukhensis*	2	1	0	0	0	NA	NA	NA	NA	NA
*M. halei*	4	1	0	0	0	NA	NA	NA	NA	NA
*M. infumata*	11	3	0.564	2	0.0017	0.0362	−0.2696	−0.113	0.094215	0.000715
*M. laciniatula*	16	5	0.833	3	0.0032	1.2695	1.2620	−0.768	0.156667	0.023146
*M. lobulata*	3	NA	NA	NA	NA	NA	NA	NA	NA	NA
*M. multispora* 1	14	2	0.143	1	0.0004	−1.1552	−1.5139	−0.595	0.168216	0.005273
*M. multispora* 2	2	2	1	1	0.0024	NA	NA	NA	NA	NA
*M. multispora* 3*	26	15	0.911	31	0.0150	−1.0675	−0.7554	−3.234	0.015233	0.007078
M. olivacea*	34	24	0.959	26	0.0092	−1.5576	**−2.6561**	−19.249	0.018858	0.000997
*M. olivaceoides* 1	8	1	0	0	0	NA	NA	NA	NA	NA
*M. olivaceoides* 2	2	1	0	0	0	NA	NA	NA	NA	NA
*M. poeltii*	3	2	0.667	3	0.0050	NA	NA	NA	NA	NA
M. septentrionalis*	31	9	0.501	16	0.0048	−1.7399	−2.4269	−2.221	0.169296	0.304035
*M. subelegantula*	10	3	0.378	5	0.0025	**−1.7411**	−2.1790	0.477	0.285432	0.039008
*M. subexasperata*	2	2	1	7	0.0141	NA	NA	NA	NA	NA
*M. subolivacea* 1	9	7	0.917	15	0.0100	−0.4538	−0.7083	−1.23	0.110969	0.046933
*M. subolivacea* 2*	76	43	0.945	30	0.0107	−0.9541	−2.2016	−20.111	0.008245	0.00313
*M. trabeculata*	10	3	0.6	5	0.0056	0.8301	0.7679	2.146	0.102222	0.043291
*M. ushuaiensis* Argentina	1	1	0	0	0	NA	NA	NA	NA	NA
*M. ushuaiensis* Chile	2	2	1	3	0.0075	NA	NA	NA	NA	NA

*n* = sample size, *h* = number of haplotypes, Hd = haplotypic diversity, S = number of segregating (polymorphic) sites, π = nucleotide diversity, Fs = Fu’s F statistic, RI = raggedness index, SSD = sum of squared deviations. **Bolded** values indicate significant Tajima D, Fu’s F statistic, RI and SSD values.

Although both Tajima’s *D* and Fu’s *F* statistic values were negative in most cases (Table 4), Tajima’s *D* statistic was significant (*P* < 0.05) and negative for *M. elegantula* (−2.137) and *M. subelegantula* (−1.741); and Fu’s *F* statistic was significant and negative for *M. elegantula* (−3.011) and *M. olivacea* (−2.6561). The mismatch distributions generally showed relatively unimodal patterns for taxa commonly producing vegetative diaspores (*M. elegantula*, *M. exasperatula*, *M. infumata*, and *M. laciniatula*), and mismatch distributions largely appeared multimodal for taxa producing ascomata (*M. exasperata*, *M. multispora* 3, *M. septentrionalis*, *M. subolivacea* 1, *M. subolivacea* 2, and *M. trabeculata*) (Additional file 3). No tests were carried out for *M. exasperata* Spain, *M. gomukhensis*, *M. halei*, *M. elegantula* Spain, *M. lobulata*, *M. multispora* 2, *M. olivaceoides* 1, *M. olivaceoides* 2, *M. poeltii*, *M. subexasperata*, *M. ushuaiensis* Argentina, and *M. ushuaiensis* Chile, due either to their lack of polymorphisms or small sample sizes.

**Figure 3:**
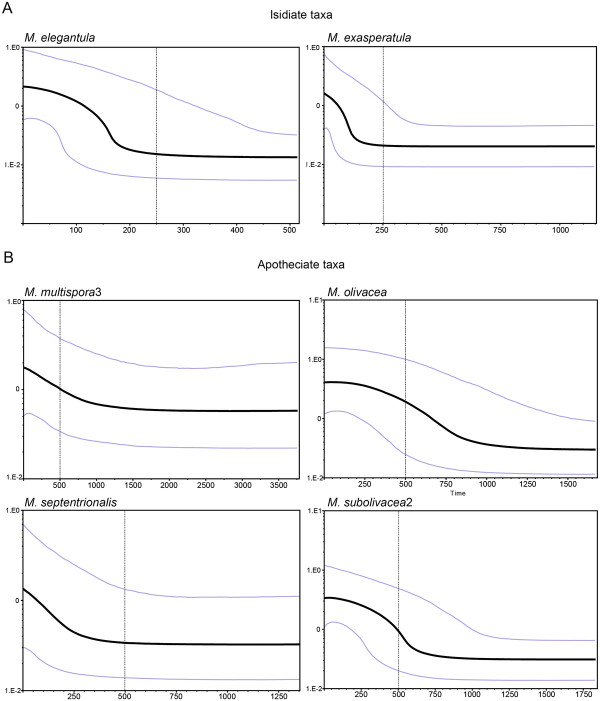
**Bayesian skyline plots for (A) two *Melanohalea *species commonly propagating via vegetative diaspores (*M. elegantula *and *M. exasperatula *) and (B) four sexually reproducing lineages (*M. multispora *3, *M. olivacea *, *M. septentrionalis *, and *M. subolivacea *2).** The solid black line represents the median value for the log of the population size (log *N*e) and the grey lines represent the upper and lower 95% credible intervals. The *x-*axis measures times in thousands of years. Generation times are not known for Melanohalea species and population sizes (y-axis) only represent relative changes.

The effective population sizes and demographic trends estimated by the Bayesian skyline plot (BSP) analyses for *M. elegantula*, *M. exasperatula*, *M. multispora* 3, *M. olivacea*, *M. septentrionalis*, and *M. subolivacea* indicated population size increases during the Pleistocene for the four sexually reproducing species and two taxa commonly producing vegetative diaspores (Figure 3A-B). However, BSP plot analyses suggest population expansion predated the end of the LGM in all sampled species. Our data suggest that the overall increase of the two taxa commonly producing vegetative diaspores occurred much more recently than the expansions of the lineages strictly reproducing through sexual propagation (i.e. taxa that do not produce vegetative diaspores), with the exception of *M. septentrionalis* (Figure 3A-B). Based on the mutation rate of 0.033 s/s/Myr for the ITS marker, population growth started at approximately 200,000 years before present (BP) in the sampled isidiate taxa, *M. elegantula* and *M. exasperatula* (Figure 3A). In contrast, population expansion for the sexually reproducing taxa *M. multispora*, *M. olivacea*, and *M. subolivacea* appears to have started approximately 750,000 to 100,000 years BP (Figure 3B). However, the BSP for the apotheciate taxon *M. septentrionalis* suggests a more recent expansion, relative to the other apotheciate taxa (Figure 3B).

## Discussion

### Concatenated gene tree and multilocus divergence estimates

In this study, we estimated divergence times for the lichen-forming fungal genus *Melanohalea* using Parmeliaceae-specific substitution rates for three DNA regions. McCormack et al. [18] suggested that estimating divergence times using a multilocus species tree approach is especially relevant for investigating taxonomic groups with poor fossil representation, and here we provide the first implementation of calibrated coalescent-based species-tree divergence time estimates for lichen-forming fungi.

Similar to a previous study [18], we found that divergence times varied greatly among estimates from concatenated gene tree and multilocus species-tree approaches. A comparison of the divergence estimates from the concatenated gene tree versus multilocus coalescent-based species-tree showed a consistent pattern of more recent divergence times estimated from the species trees approach, regardless of node age (Figure 2, Table 3). Although gene trees will necessarily overestimate divergence times in recent speciation events [60], in this study we found the greatest disparity in estimates at deeper relationships, differing by over 8 million years at some nodes (Table 3). Additionally, we found that the multilocus coalescent-based species tree approach consistently provided narrower 95% confidence intervals than the concatenated gene tree approach (Table 3).

Topologies derived from concatenated datasets are not necessarily equivalent to species trees [61-65]. In addition to inferring incorrect relationships and estimates of support, using concatenated gene trees for estimating divergence times will likely produce overestimates for the divergence events [18,66]. In contrast, methods incorporating a full probabilistic coalescent framework, such as the *BEAST analysis implemented herein, have been proposed to provide more biologically realistic estimates because multiple gene trees provide independent realizations of divergence history, accounting for mutational and coalescent stochasticity [18].

### Pre-Pleistocene divergence

We estimated that the *Melanelixia- Melanohalea* split occurred during the Eocene, with stem node age estimates of 41.6 or 35.2 Ma in the concatenated and multilocus species tree approaches, respectively (Figures 1, 2, Table 3). A previous study suggested that the radiation of major parmelioid genera started at the end of the Eocene [12], and our estimates support a similar pattern of divergence for the *Melanelixia* and *Melanohalea* split. Our data suggest that the radiation of *Melanohalea* began either during the Oligocene, or the transition at the Oligocene – Miocene boundary, with crown node age estimates of 30.5 Ma and 23.7 Ma in the concatenated and multilocus species tree approaches, respectively (Figures 1, 2, Table 3). The date from the concatenated analysis is approximately five million years older than that presented in Amo de Paz et al. [12], but this is most likely due to our inclusion of the early diverging South American endemic *M. ushuaiensis* sensu lato, which was absent from their analysis.

Our results also suggest that most major speciation events within *Melanohalea* occurred during the Miocene and Pliocene, and were likely the result of complex patterns associated with new habitats formed during Miocene orogeny events, major climatic changes, and global shifts in vegetation. The Miocene was a time of major climatic and vegetative changes worldwide, especially in the northern Hemisphere, including major tectonic activity and orogeny [18,67-70]. Increasing aridity in the middle Miocene (15–8 Ma; [71,72]) resulted in woodlands giving way to more open habitats and grasslands [69,73-75]. However, given the incredible dispersal capacity of some *Melanohalea* species, and other fungi within Parmeliaceae (e.g. [12,33,34,43]), inferring the geographic origin of lineages and associated temporal biogeographic factors remains challenging.

Distributions of most *Melanohalea* species are largely determined by contemporary ecogeographical factors, and most species have largely filled their potential distributions in the northern Hemisphere [43]. In general, the distributions are in high concordance with those found in vascular plants and can be widely explained by contemporary ecogeographical factors [76]. Although the distribution of lichenized fungal species is limited by the availability of the appropriate substrata, other unrecognized dispersal barriers appear to have limited the dispersal of some *Melanohalea* species. For example, the amphiatlantic distribution of *M. exasperata* and the strictly European distribution of *M. laciniatula* remain unexplained. Although we currently do not know the origin of the distribution patterns in these two species, incomplete spread into North America, particularly western North America, cannot be excluded [43].

Interestingly, within the morphologically cryptic *M. multispora* and *M. ushuaiensis* species complexes, species-level lineages also appear to have diversified largely during the Miocene (Figures 1, 2). The two defined *M. olivaceoides* sensu lato (s.l.) lineages [59] were not recovered as sister taxa and their most recent common ancestor (MRCA) was estimated over 20 Ma (Figures 1, 2). Similarly, the morphologically similar taxa *M. elegantula* and *M. infumata* are found in separate major clades within *Melanohalea*, with the MRCA estimated to have been over 20 Ma in the dated coalescent-based species tree (Figure 2). These results suggest that in some cases, i.e. *M. multispora* s.l and *M. ushuaiensis* s.l, taxonomically diagnostic characters, including number of spores and isidia morphology may be maintained among divergent lineages for millions of years. In other cases, similar phenotypic characters, among non-sister taxa, including reproductive strategies (i.e. the production of isidia, soredia, and apotheica), suggest the potential for convergent evolution due to similar selective pressures among distinct lineages inhabiting similar environments [7,77,78].

Climatic changes during the Pleistocene are hypothesized as one of the foremost contributors to biological diversity (e.g. [18,53-55]). However, in this study, we show that species divergence during Pleistocene glacial cycles was restricted to only a limited number of clades. Of the morphologically indistinguishable species-level lineages recognized in [59], only *M. subolivacea* sensu lato and *M. elegantula* sensu lato appear to have diversified during the Pleistocene. However, additional supporting evidence, such as morphology, geographic range, ecological preference, chemistry, etc., will be required to establish robust species boundaries among the recently derived lineages.

Our data suggests that two other clades also diversified during the Pleistocene, one containing the sorediate taxa *M. gomukhensis*, *M. olivaceoides *1 and the isidiate taxon *M. poeltii*; and the second containing the apotheciate taxa *M. lobulata* and *M. subexasperata* and the sorediate taxon *M. olivaceoides *2. In both clades, these species are restricted to Asian highlands (i.e. Tibetan Plateau and northern India) and northern latitudes of western North America. This unique distribution pattern found in two distinct clades impacted by Pleistocene divergence provides circumstantial evidence of transcontinental dispersal from the Asian highlands to the New World through Beringia. Studies have demonstrated that Beringia has served as a biological corridor for the dispersal of a variety of organisms (e.g. [79-83]), and we hypothesize that glacial refugia in Beringia during the Pleistocene have likely played an important role in transcontinental dispersal and diversification for some lichenized fungi, including *Melanohalea*.

### Demographic histories

During the Pleistocene, glacial advances affected the physical and biological environments of the Northern Hemisphere [84-86]. The more traditional methods for inferring population growth used in this study (i.e., Fu’s *F*, and Tajima’s *D*, and mismatch distribution) predict population expansion unambiguously for only *M. elegantula*, although Fu’s *F* and Tajima’s *D* were negative but not significant for other taxa, including lineages assessed using BSP analyses (*M. exasperatula*, *M. multispora *3, *M. olivacea*, *M. septentrionalis*, and *M. subolivacea *2). In our study mismatch distributions generally showed relatively unimodal patterns for taxa commonly producing vegetative diaspores (isidiate taxa), and mismatch distributions largely appeared multimodal for taxa producing ascomata (apotheciate taxa) (Additional file 3). Although a multimodal mismatch distribution is generally interpreted as evidence for population stability [87], BSP analyses of apotheciate taxa with multimodal mismatch distributions suggested Pleistocene population expansion of the four tested lineages. Multimodal distributions that fit sudden-expansion models can also be an indication of infraspecific structuring [88], a pattern consistent with the phylogenetic sub-structuring present in the many apotheciate taxa in the ITS topology (Additional file 2). Additional research investigating infraspecific population structure in fertile *Melanohalea* species and associated demographic histories will be essential to assess the reliability of the demographic histories inferred in this study.

BSP analyses suggest that population expansions predate the LGM in apotheciate taxa (*M. multispora *3, *M. olivacea*, *M. septentrionalis*, and *M. subolivacea* 2), as well as those propagating via vegetative diaspores (*M. elegantula* and *M. exasperatula*). Our results indicate that at least some *Melanohalea* populations were able to expand during Pleistocene glacial cycles, albeit with apparently different demographic histories. Similar to other organisms [54,89,90], these data suggest that Pleistocene glaciations were not inherently unfavorable or restrictive for some *Melanohalea* species. However, the relative impact of interglacial periods on population expansion in *Melanohalea* remains unclear.

With the exception of *M. septentrionalis*, apotheciate taxa evaluated here appear to have expanded over 700 Ka, suggesting a similar response to Pleistocene climatic factors among both broadly distributed (*M. olivacea*) and geographically restricted species (*M. multispora *3 and *M. subolivacea *2). The differences in the timing of population expansions seen between *M. septentrionalis* and other apotheciate species may be related to ecology as the treeline migrated north [91]. *Melanohalea septentrionalis* is essentially a boreal species, while outpost populations of *M. olivacea* south of the boreal zone are markedly more common than in *M. septentrionalis*[43]*. Melanohalea multispora *3 and *M. subolivacea *2 are essentially western North American species, commonly found in dry forests [92]. The contemporary distributions patterns and results from the BSP analyses suggest that climatic changes and associated shifts in vegetation in non-glaciated ecosystems during the Pleistocene had a significant impact on population demographics of fertile *Melanohalea* species.

Although it has been proposed that the contemporary distribution and abundance of common isidiate *Melanohalea* species is a result of recent expansion after the LGM and subsequent anthropogenic disturbances [48-51,57,76], BSP analyses of vegetatively propagating species (*M. elegantula* and *M. exasperatula*) suggest synchronous population expansion ca. 200 Ka, with 95% confidence interval providing strong evidence that this expansion predates the LGM (Figure 3). BSP analyses indicate that the beginning of the population expansion of these taxa coincides with the Saalian Pleniglacial cycle (ca. 182 Ka) and continued the expansion into the Weichselian glacial cycle (beginning ca. 110 Ka; [93]). Both isidiate taxa have broad ecological amplitude and are commonly found in formerly glaciated areas, as well as lower elevation sites, but are also found in other nutrient-rich habitats [43].

Nimis [58] argued that the distribution of Holarctic lichens is likely determined by general ecology rather than by their reproductive strategy alone. The latter argument supports the assumption that the distribution of *Melanohalea* within the Holarctic today widely reflects their biological constitution, rather than their geographic origin or dispersal strategy. However, contemporary distributions of isidiate taxa, *M. elegantula* and *M. exasperatula*, and apotheciate taxa, *M. multispora* 3, and *M. subolivacea* 2, largely overlap in western North America, and these species co-occur to varying degrees in some habitats. In spite of the fact that these species share similar distributions and habitats in some regions, BSP analyses suggest that lineages sharing reproductive strategies also share similar demographic histories, with population expansions in apotheciate lineages largely predating expansions in isidiate lineages. Whether these similarities are due to reproductive strategy-dependent fitness advantages, availability of the appropriate photosynthetic partner or fine-scale ecological niches remains unknown and merits future investigation.

Caution must be taken with the results from the BSP analyses presented here due to the potential limitations of using genetic data from the ribosomal tandem repeat and potential violations of the assumption of panmixia in lineages reproducing largely via vegetative diaspores. The selection of genetic loci for inferring demographic histories is not always straightforward (reviewed in [94]). Mitochondrial markers have been the locus of choice in skyline-plot analyses of animal populations [94-97]. However, within Parmeliaceae, mitochondrial markers generally have very low intraspecific variation [28,31,34,36]. Additionally, introns and paralogous copies of other commonly used mitochondrial markers have been found in fungi (reviewed in [98]). Although previous studies have found evidence of multiple ITS sequence types within individuals of several fungal species, including lichenized fungi [99-101], intragenomic ribosomal variation has not been reported in Parmeliaceae. Multiple studies have confirmed the utility of variable markers within the ribosomal cistron (i.e., ITS and IGS) for species and population-level studies in lichenized ascomycetes [5,8,37,102-108]. Furthermore, the ITS region has a high mutation rate and is the most variable marker commonly used in Parmeliaceae [7,8,28,36,37]. In this study we found that the substitution rate was over two times higher than that of the sampled protein-coding loci (*MCM*7, *RPB*1, and *RPB*2) and nearly five times higher than that of the sampled mitochondrial marker (mtSSU).

We are aware of potential limitations of using ribosomal markers to infer demographic histories, but we consider this a good “first pass” marker with which to investigate population demographics in lichenized fungi. While additional studies will be required to confirm the utility of the ITS marker for inferring demographic histories of lichenized ascomycetes using Bayesian skyline analyses, our study provides a valuable “hypothesis generating” approach for assessing historical factors driving population dynamics in lichen-forming fungi.

## Conclusions

In this study we provide a comparison of dated divergence time estimates between concatenated gene trees and a calibrated multilocus species tree for the lichen-forming fungal genus *Melanohalea*. Our results showed a consistent pattern of more recent divergence times estimated from the coalescent-based species tree approach, relative to the concatenated gene tree analysis. Although earlier divergence times were estimated across the species tree topology, relative to dates estimated from the concatenated gene tree, we found the greatest disparity between methods at deeper nodes. This discrepancy clearly has important consequences for understanding factors driving diversification, extinctions, and biogeographic patterns. Given the results of this study, we propose that additional care must be taken when attempting to place divergence events within a temporal context by including estimates from calibrated coalescent-based species tree methods. Results from both divergence dating methods implemented herein indicate that most diversification within *Melanohalea* occurred during the Miocene and Pliocene, and was likely the result of habitat changes due to mountain uplift, major climatic changes (aridification) and associated shifts in vegetation patterns. This timeframe was also a period of significant mountain uplift, including major phases in the Andes [109], Alps [110], and Himalayas [111]. However, the temporal concordance of these diversification events with major vegetation changes in other geographical regions suggests that a more global environmental driver likely contributed to diversification in lichen-forming fungi in Parmeliaceae [12]. However, Pleistocene glacial cycles appear to have played a limited role in the diversification of some taxa in the Asian highlands and northern latitudes of North America and morphologically two cryptic lineages in western North America and Europe. In addition, we provide evidence for population expansions of common *Melanohalea* species during the Pleistocene. Our data provide evidence that the abundance of *M. elegantula* and *M. exasperatula* is likely not the result of anthropogenic factors. Rather, the population expansion of these common species coincides will the beginning of the Saalian glacial period. Although the timing of population expansions of distinct reproductive strategies predates the LGM, lineages commonly reproducing with vegetative diaspores expanded more recently (ca. 200 Ka) than most strictly sexually reproducing species (> 750 Ka). These data suggest that Pleistocene glaciations were not inherently unfavorable or restrictive for some *Melanohalea* species.

## Methods

### Taxon sampling

In the present study we obtained sequence data from a total of 487 *Melanohalea* specimens (Additional file 1). Overall, 18 of the 22 species listed for the genus in Index Fungorum (http://www.indexfungorum.org/) and seven previously unrecognized species-level lineages [59], awaiting formal description, were represented in this study. In this study nomenclature of undescribed species-level lineages follows arbitrary names in [59]. In order to infer changes in effective population sizes of common *Melanohalea* species over time, our sampling focused on multiple populations throughout the known distributions of four sexually reproducing species and two taxa commonly producing vegetative diaspores. For the sexually reproducing taxa, we selected two species with distributions restricted to western North America, *M. multispora *3 (26 specimens) and *M. subolivacea *2 (77); and two species with broader geographic distributions, *M. olivacea* (35) and *M. septentrionalis* (31). We sampled the broadly distributed isidiate taxa *M. elegantula* (100) and *M. exasperatula* (105) across their known distributions, representing common taxa reproducing vegetatively. *Melanelixia californica* was used as the outgroup for phylogenetic analyses [12,28,39].

### DNA isolation, polymerase chain reaction (PCR) amplification and sequencing

Total genomic DNA was extracted from a small section of thallus material using the Prepease DNA Isolation Kit (USB, Cleveland, Ohio, USA), following the plant leaf extraction protocol. For all samples we sequenced the internal transcribed spacer region (ITS, ~500 bp). For a subset of these samples (Additional file 4), representing sampled genetic diversity identified from ITS sequence data, we amplified fragments from the nuclear ribosomal large subunit (LSU, ~530 bp), mitochondrial small subunit (mtSSU, ~800 bp), and three low-copy protein coding markers, *RPB*1 (~775 bp), *RPB*2 (~716 bp) and *MCM*7 (~514 bp). We developed new taxon-specific primers for the ITS, *RPB*2, and *RPB*1 in order to improve PCR specificity and efficiency in some cases. All primers used to amplify and sequence loci used in this study are provided in Table 5. PCR amplifications were conducted in 25 μL reactions. In some cases where standard PCR failed to amplify target loci, we used Ready-To-Go PCR Beads (GE Healthcare) following manufacturer’s recommendations with markedly improved success. PCR cycling parameters for amplifying the ITS and LSU loci followed [31]; cycling parameters for the RPB1, RPB2, and *MCM*7 fragments followed the *MCM*7 protocol described by [112]. PCR products were quantified on 1% agarose gel and stained with ethidium bromide. PCR fragments were cleaned using either the PrepEase PCR Purification Kit (USB, Cleveland, Ohio, USA) or ExoSAP-IT (USB, Cleveland, Ohio, USA), following manufacturers’ instructions. Complementary strands were sequenced from cleaned PCR products using the same primers used for amplifications. Sequencing reactions were performed using BigDye v3.1 (Applied Biosystems, Foster City, CA, USA). Products were run on an ABI 3730 automated sequencer according to recommended protocols (Applied Biosystems) at the Pritzker Laboratory for Molecular Systematics at the Field Museum, Chicago, IL, USA.

**Table 5:** All primers used for PCR amplification and sequencing in this study

**Marker**	**Primer name**	**Forward primer sequence**	**Annealing temperature (°C)**	**Reference**
**ITS**	ITS1F	5’-CTTGGTCATTTAGAGGAAGTAA-3’	55-60	[113]
	ITS_Mel_F	5’- TGCTTTGGCGGRYCYYRRG-3’	55-60	This study
	ITS4	5’-TCC CCGCTTATTGATATGC-3’	55-60	[114]
	ITS4A	5’- CGCCGTTACTGGGGCAATCCCTG-3’	55-60	[115]
	ITS4	5’-TCCTCCGCTTATTGATATGC-3’	55-60	[114]
**LSU**	Al2R	5’-GCGAGTGAAGCGGCAACAGCTC3’	55-60	[116]
	LR3	5’-CCGTGTTTCAAGACGGG-3’	55-60	Vilgalys unpublished
**mtSSU**	mrSSU1	5’-AGCAGTGAGGAATATTGGTC-3’	55-60	[117]
	mrSSU3R	5’-ATGTGGCACGTCTATAGCCC-3’	55-60	[117]
***MCM7***	Mcm7-709for	5’-ACIMGIGTITCVGAYGTHAARCC-3’	56	[112]
	Mcm7-1348rev	5’-GAYTTDGCIACICCIGGRTCWCCCAT-3’	56	[112]
	X_Mcm7_F	5’-CGTACACYTGTGATCGATGTG-3’	56	[118]
	X_Mcm7_R	5’- GTC TCC ACG TAT TCG CAT TCC-3’	56	[118]
**RPB1**	gRPB1-A for	5’-GAKTGTCCKGGWCATTTTGG-3’	54-56	[119]
	fRPB1-C rev	5’-CCNGCDATNTCRTTRTCCATRTA-3’	54-56	[119]
	RPB1_MH_F	5’-ACGTCGCCGAGACCCHAARA-3’	54-56	This study
**RPB2**	RPB2-6F	5’-TGGGGKWTGGTYTGYCCTGC-3’	50-56	[120]
	fRRPB2-7cR	5’-CCCATRGCTTGYTTRCCCAT-3’	50-56	[120]
	RPB2_MH_F	5’-ACAGTCGGTACWCCCAGYGAGCC-3’	50-56	This study
	RPB2_MH_R	5’-TGCCCATAGCCGATTGGTAYGTATT-3’	50-56	This study

### Sequence alignment

 We assembled and edited sequences using the program Sequencher v3.1.1 (Gene Codes Corporation, Ann Arbor, MI) and Geneious v5.4 [121]. Sequence identity was confirmed with ‘megaBLAST’ search in GenBank [122]. Sequences were aligned using the program MAFFT v6. For the LSU, *MCM7*, *RPB*1, and *RPB*2 markers we implemented the G-INS-I alignment algorithm, ‘1PAM / K = 2’ scoring matrix, and offset value = 0.8, with the remaining parameters set to default values; for the ITS we used the same G-INS-I alignment algorithm, ‘1PAM / K = 2’ and scoring matrix, and offset value = 0.1; and for the mtSSU we used the E-INS-I alignment algorithm, ‘20PAM / K = 2’ scoring matrix, and offset value = 0.0. For the mtSSU MAFFT alignment we also used the program Gblocks v0.91b [123,124] to remove regions of alignment uncertainty, using options for a “less stringent” selection on the Gblocks web server (http://molevol.cmima.csic.es/castresana/Gblocks_server.html).

### Molecular dating

In this study we provide estimates of divergence events for species-level lineages presented in [59]. In order to obtain divergence date estimates for *Melanohalea*, we used two Bayesian approaches implemented in BEAST v1.6.1 [23]. We estimated divergence times under a gene-tree framework from the concatenated data matrix (i.e., ITS, nuLSU, mtSSU, *MCM*7, *RPB*1, and *RPB*2) and a multi-locus species tree approach.

For the divergence estimates in the concatenated gene tree approach we used a user-specified chronogram as the starting tree, rather than a randomly generated tree (e.g. [12]). To generate the starting topology, we conducted a ML analysis of the six-locus dataset (ITS, nuLSU, mtSSU, *MCM*7, *RPB*1, and *RPB*2) using locus-specific model partitions in RAxML v7.2.7 [125,126], and all loci were treated as separate partitions. We used the GTRGAMMA model, which includes a parameter (Γ) for rate heterogeneity among sites and chose not to include a parameter for estimating the proportion of invariable sites [125,126]. A search combining 200 separate maximum likelihood searches to find the optimal tree was conducted. The ML topology obtained from the RAxML analysis was converted to an ultrametric tree using nonparametric rate smoothing (NPRS) implemented in TreeEdit v.10a10 [127] for the BEAST analyses. In BEAST, the molecular dataset was analyzed with unlinked substitutions models across the loci, a birth-death model was used as a prior for the node heights, and a relaxed clock model (uncorrelated lognormal) for each partition. In the absence of relevant fossil evidence for *Melanohalea*, we used molecular evolution rates for *RPB*1, nuLSU, and mtSSU loci recently reported for Parmeliaceae [12]. While we used the *Melanohalea*-specific rate reported for the *RPB*1 locus (1.69 × 10^-9^ s/s/y), *Melanohalea*-specific rates were not available for the nuLSU and mtSSU loci [12]. Therefore, we used rates estimated for Parmeliaceae (*Protoparmelia* excluded) for the nuLSU (0.70 × 10^-9^ s/s/y) and mtSSU (0.69 × 10^-9^ s/s/y) to estimate the time to the most recent common ancestor (mrca) for all clades [12]. Substitution rates for the ITS, *MCM*7, and *RPB*2 markers were co-estimated along the run under a uniform prior, relative to the previously published rates for the nuLSU, mtSSU, and *RPB*1 loci. The rate in each branch was drawn from either an exponential or lognormal distribution. Two independent analyses were run for 50 million generations and parameter values were sampled every 1000 generations. The output from each analysis was visualized using Tracer v1.5 [128] to assess convergence and effective sampling size (ESS) of sampling parameters. Convergence was also assessed in AWTY [129] to ensure that standard deviations of split frequencies between runs approached zero and visualize split probabilities. Based on these results the first 12.5 million generations were discarded as burn-in, and remaining samples were summarized as a maximum clade credibility tree with mean divergence times and HPD intervals of age estimates using TreeAnnotator v.1.6.1 [130].

Gene trees, including phylogenies estimated from concatenated sequence data, can overestimate divergence times because they do not correct for genetic divergence that predates speciation [18]. Species tree methods incorporating the process of gene lineage coalescence likely provide a more biologically realistic framework for dating divergence events [18]. For comparison to concatenated-based divergence estimates, we thus also estimated divergence dates using a coalescent-based species-tree approach. We used the hierarchical Bayesian model *BEAST, implemented in program BEAST v1.6.1 [131], to estimate a species tree from lineages defined in [59]. *BEAST estimates the species tree directly from the sequence data, and incorporates the coalescent process, uncertainty associated with gene trees, and nucleotide substitution model parameters [131]. Models of DNA sequence evolution for each marker were selected with the program jModeltest v0.1 [132], using the AIC criterion. Implementing an uncorrelated relaxed lognormal clock [133], we selected a birth-death model for the species tree prior; the population size model was set to piecewise linear and constant root. Molecular evolution rates for the nuLSU, mtSSU, and *RPB*1 loci were as defined above. Default values were used for remaining priors. Two independent MCMC analyses were run for a total of 100 million generations (sampling every 2000 steps and excluding the first 25 million generations of each run as burn-in). Convergence was assessed by ensuring that standard deviations of split frequencies between runs approached zero, visualizing split probabilities in AWTY [129], and comparing summarized tree topologies from separate runs. We assessed the effective sample sizes (ESS) of parameters of interest to ensure sample sizes were all above 200. Posterior probabilities of nodes were computed from sampled trees after burn-in.

### Molecular Diversity and population demographics

We used the program DnaSP v4.50 [134] to calculate estimates of genetic diversity (including number of haplotypes, *h*; haplotypic diversity, *Hd*; number of polymorphic sites, *S*; and nucleotide diversity, *π*) from ITS sequence data for each species. To detect possible departures from a constant population size that could be interpreted as a result of a past demographic expansion, we calculated the Fu’s *F* statistic [135] and Tajima’s *D*[136] for each species represented by ≥ 9 individuals. Significant and negative Tajima’s *D* and Fu’s *F* statistic values are indicative of possible population expansion, and positive values of these sample statistics provide evidence of a recently bottlenecked population or diversifying selection. These statistics were calculated in DnaSP, and significance was determined using the coalescent process implemented in DnaSP (1000 replicates). Mismatch frequency histograms were also plotted for each species represented by ≥ 9 individuals in DnaSP to determine whether clades exhibited evidence of spatial range expansion or a stationary population history. A smooth bell-shaped curve suggests either population expansion or spatial range expansion, whereas a multimodal curve represents a long history *in situ*. A raggedness index (RI, sum of the squared difference between neighboring peaks) and sum of squared deviations (SSD) between observed and expected mismatch were calculated in Arlequin v3.5 [137] using a parametric bootstrap approach (1000 replicates) to test for significance.

Although Tajima’s *D*, Fu’s *F* and mismatch distributions can provide insight into whether or not populations have undergone changes in population size, they do not provide information about the shape of the population growth over time. Here changes in species-specific demographic histories over time were assessed using Bayesian skyline analysis [138] in BEAST v1.6.1 [23] using the ribosomal ITS marker. A skyline plot is a model of population size fitting a wide range of demographic histories, and if a molecular clock rate is known for the locus in question, the model can be used to put demographic events into a historical context [138]. The Bayesian skyline model uses standard Markov chain Monte Carlo (MCMC) sampling procedures to estimate a posterior distribution of effective population size through time from gene sequences, given a model of sequence evolution. We selected six lineages characterized in [59] from which we have obtained sequences from specimens collected throughout their known distributions to minimize the violation of several simplifying assumptions [94,139]. Sexually reproducing taxa were represented by *M. multispora *3 (26 specimens), *M. olivacea* (35), *M. septentrionalis* (31), and *M. subolivacea *2 (77); and isidiate taxa included *M. elegantula* (100 specimens) and *M. exasperatula* (105). Although taxa that have been thought to reproduce primarily via asexual diaspores may violate the assumption of panmictic populations, sexual reproductive structures (apothecia) are occasionally found on some specimens of *M. elegantula* and *M. exasperatula* and may play an important role in population-wide gene flow [140]*.* Furthermore, recent data suggests that reproductive strategies in lichen-forming fungi traditionally considered to reproduce vegetatively are more complex than previously recognized [7,141].

Because systematic rate heterogeneity is not expected in intraspecific data, we used a strict molecular clock model with a fixed rate of 3.30 × 10^−9^ substitutions/site/year, obtained from the molecular dating analysis described above. Two independent analyses were run for 50 million Monte-Carlo-Markov-Chain generations, sampling parameter values every 1000 generations, using the Bayesian Skyline tree model allowing five discreet changes in population size with linear growth between population size change events was applied with a UPGMA generated tree as the starting point. We used the GTR + G + I model of nucleotide substitution without partitioning the ITS1, 5.8S, and ITS2 regions. We combined two independent runs and all ESS were > 200. We used Tracer v1.5.1 to analyze combined runs for each species and generate skyline plots.

## Competing interests

The authors declare that they have no competing interests.

## Authors’ contributions

HTL conceived the research and with TLE obtained funding. HTL, SDL, and TLE contributed with the conceptual development of the work. SDL, PKD, TLE, and HTL collected samples in the field. SDL and PKD performed the laboratory work. SDL performed data analyses and drafted the manuscript. All authors read and approved of the final version of the manuscript.

## Supplementary Material

Additional file 1 **Collection information for all *Melanohalea *specimens included in the present study.** Click here for file

Additional file 2 **Maximum likelihood ITS gene tree of the 487 sampled *Melanohalea *specimens.** Bootstrap support indicated at nodes, and operational taxonomic units in red text indicate 138 *Melanohalea* specimens selected to represent sampled genetic diversity in multilocus phylogenetic reconstructions. Click here for file

Additional file 3 **Mismatch distributions observed in *Melanohalea *lineages with a minimum sample size of eight individuals. ** Black squares: simulated mismatch distributions, black triangles: observed mismatch distributions. Click here for file

Additional file 4 **Selected specimens representing sampled genetic diversity and GenBank accession numbers for the six sampled loci: nuclear ribosomal internal transcribed spacer region (ITS) and large subunit (nuLSU), mitochondrial small subunit (mtSSU), and protein-coding makers *MCM *7, *RPB *1, and *RPB *2.** Click here for file
