# Supplementary figures and images for: Miocene and Pliocene dominated diversification of the lichen-forming fungal genus Melanohalea (Parmeliaceae, Ascomycota) and Pleistocene population expansions
Source: BMC Evol Biol. 2012 Sep 11;12:176. doi: 10.1186/1471-2148-12-176 (PMC3499221; doi:10.1186/1471-2148-12-176)

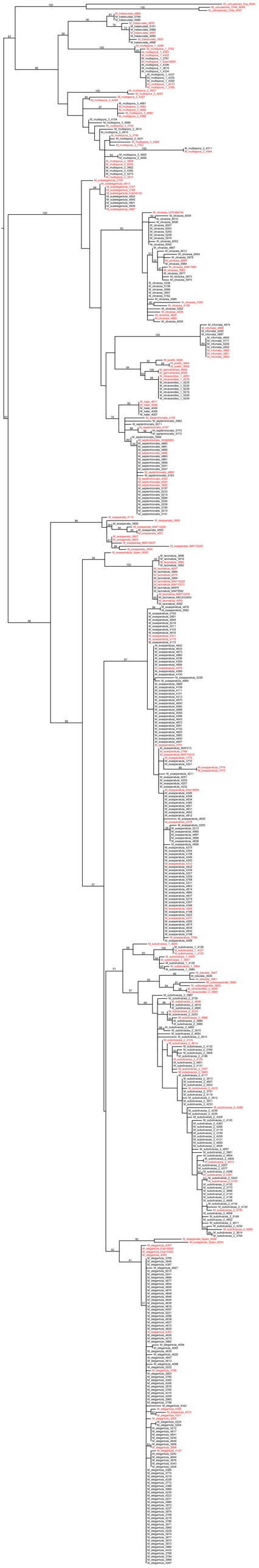

Supplement: Additional file 2 — Maximum likelihood ITS gene tree of the 487 sampled Melanohalea specimens. Bootstrap support indicated at nodes, and operational taxonomic units in red text indicate 138 Melanohalea specimens selected to represent sampled genetic diversity in multilocus phylogenetic reconstructions. [file 1471-2148-12-176-S2.pdf]
