# Supplementary material for: Miocene and Pliocene dominated diversification of the lichen-forming fungal genus Melanohalea (Parmeliaceae, Ascomycota) and Pleistocene population expansions
Source: BMC Evol Biol. 2012 Sep 11;12:176. doi: 10.1186/1471-2148-12-176 (PMC3499221; doi:10.1186/1471-2148-12-176)

M. elegantula (isidiate)

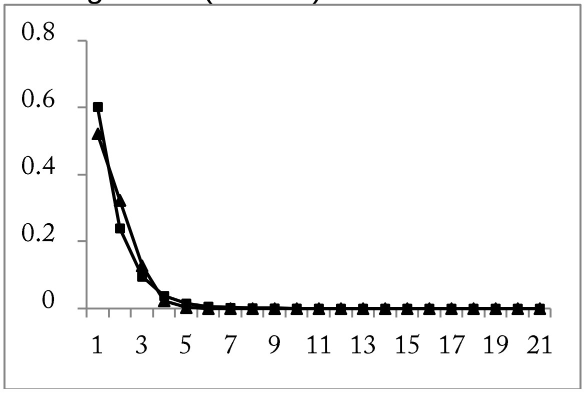

M. laciniatula (isidiate)

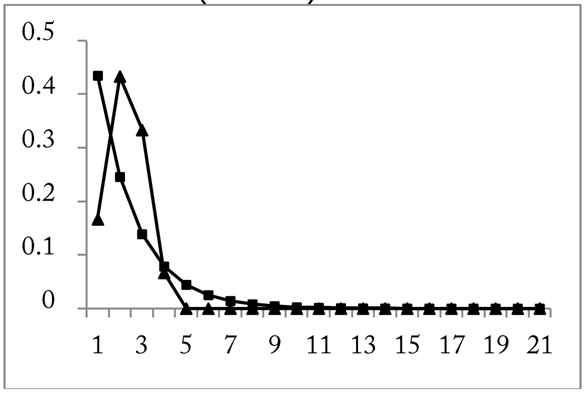

M. exasperata (apotheciate)

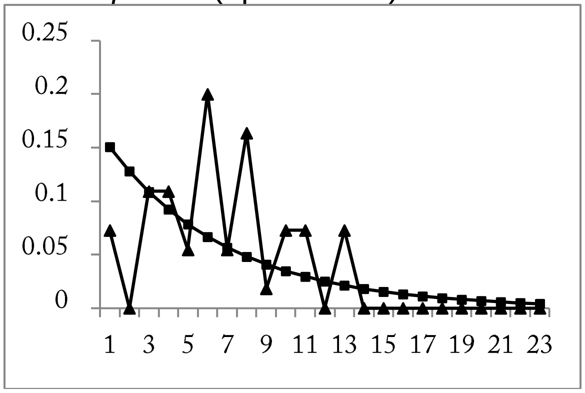

*M. multispora*1 (apotheciate)

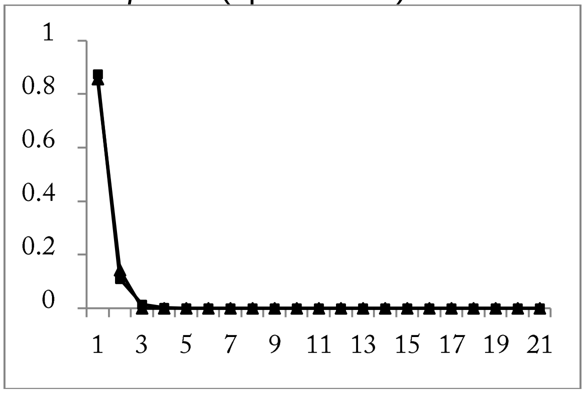

M. exasperatula (isidiate)

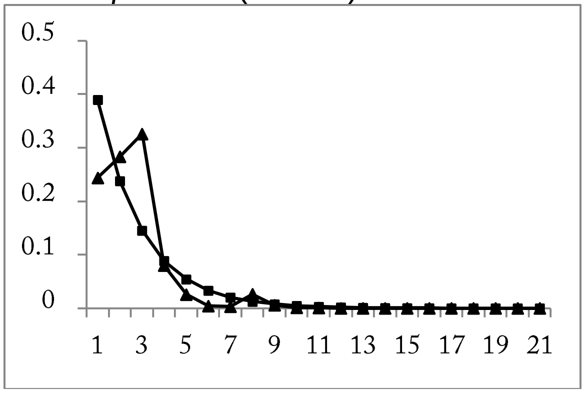

M. multispora3 (apotheciate)

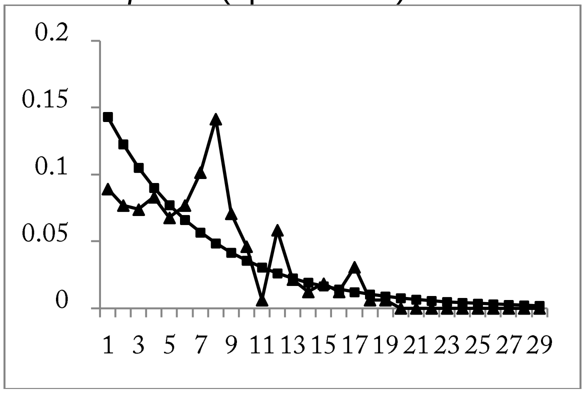

M. infumata (isidiate)

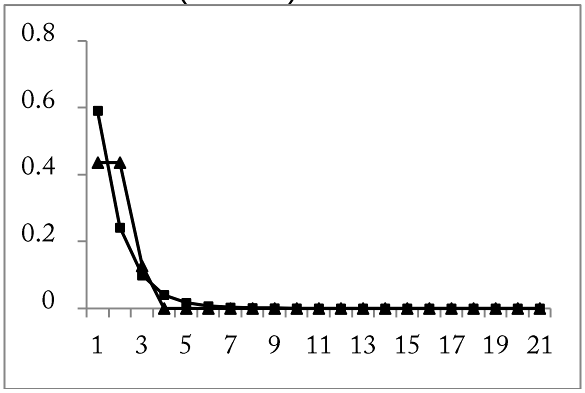

M. olivacea (apotheciate)

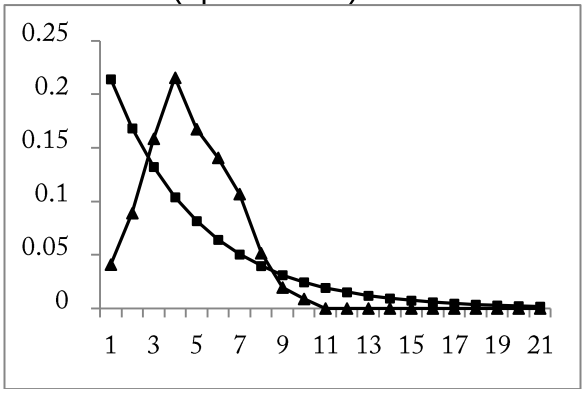

M. septentrionalis (apotheciate)

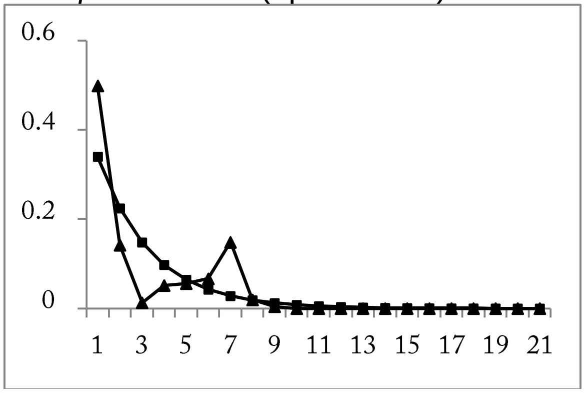

M. trabeculata (apotheciate)

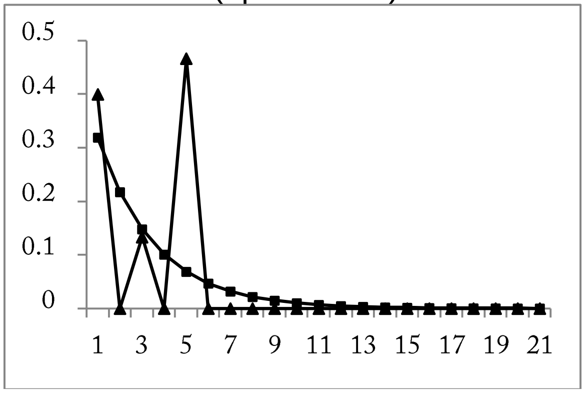

M. subelegantula (isidiate)

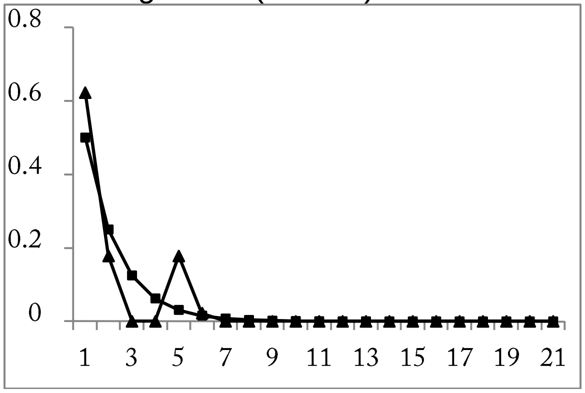

M. subolivacea1(apotheciate)

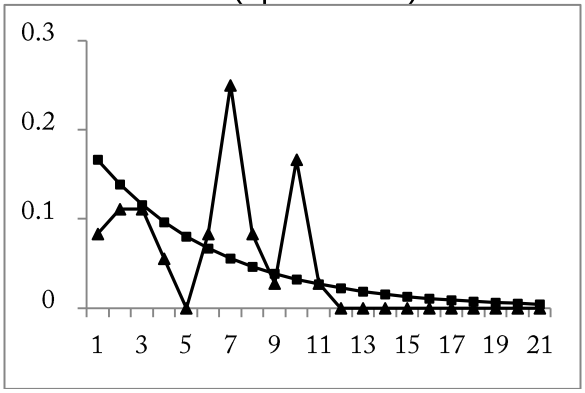

M. subolivacea2(apotheciate)

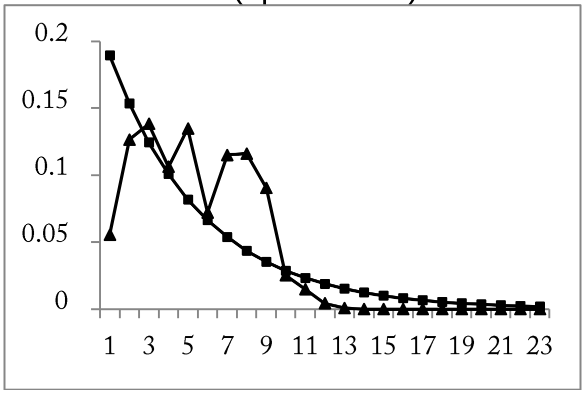

Supplement: Additional file 3 — Mismatch distributions observed in Melanohalea lineages with a minimum sample size of eight individuals. Black squares: simulated mismatch distributions, black triangles: observed mismatch distributions. [file 1471-2148-12-176-S3.pdf]
