# Supplementary material for: Miocene and Pliocene dominated diversification of the lichen-forming fungal genus Melanohalea (Parmeliaceae, Ascomycota) and Pleistocene population expansions
Source: BMC Evol Biol. 2012 Sep 11;12:176. doi: 10.1186/1471-2148-12-176 (PMC3499221; doi:10.1186/1471-2148-12-176)
Supplement: Additional file 4 — Selected specimens representing sampled genetic diversity and GenBank accession numbers for the six sampled loci: nuclear ribosomal internal transcribed spacer region (ITS) and large subunit (nuLSU), mitochondrial small subunit (mtSSU), and protein-coding makers MCM 7, RPB 1, and RPB 2. [file 1471-2148-12-176-S4.docx]

**Additional file 4.** Selected specimens representing sampled genetic diversity and GenBank accession numbers for the six sampled loci: nuclear ribosomal internal transcribed spacer region (ITS) and large subunit (LSU), mitochondrial small subunit (mtSSU), and protein-coding makers *MCM*7, *RPB*1, and *RPB*2.

| **Taxon** | **Specimen ID** | **Voucher #** | **ITS** | **nuLSU** | **mtSSU** | ***MCM*7** | ***RPB*1** | ***RPB*2** |
| --- | --- | --- | --- | --- | --- | --- | --- | --- |
| *Melanohalea elegantula* |  |  | XX000000 | XX000000 | XX000000 | XX000000 | XX000000 | XX000000 |
|  | 3745 | USA, Esslinger, 18874 (TLE) | x | x | x | x | x | x |
|  | 3905 | USA, Spribille 20670 (GZU) | x | x | x | x | x | x |
|  | 3958 | Greenland, Hansen ESH-09B.278 (TLE) | x | x | x | x | x | x |
|  | 4137 | USA, Leavitt 10-357 (F) | x | x | x | x | x | x |
|  | 4295 | USA, Leavitt 10.site10.01 | x | x | - | x | x | x |
|  | 4303 | USA, Leavitt 10.site12.01 | x | x | x | x | x | x |
|  | 4307 | USA, Leavitt 13.site13.01 | x | x | x | x | x | x |
|  | 4315 | USA, Leavitt 10.site18.01 (F) | x | x | - | x | x | x |
|  | 4317 | USA, Leavitt 10.site18.03 (F) | x | x | x | x | x | x |
|  | 4363 | USA, Leavitt 10.site45.01 (F) | x | - | - | x | x | x |
|  | Essl16362 | USA, Esslinger 16362 (TLE) | AY611120 | AY611176 | AY607834 | - | - | - |
|  | Essl16550 | USA, Esslinger 16550 (TLE) | AY611116 | AY611172 | AY607830 | - | - | - |
| *M.* *elegantula*SPAIN |  |  |  |  |  |  |  |  |
|  | 6042 | Spain, 10224 (MAF) | x | x | x | x | x | - |
| *M. exasperata* |  |  |  |  |  |  |  |  |
|  | 3665 | The Netherlands, Aptroot 68148 (F) | x | x | x | x | x | - |
|  | 3683 | Scotland, Coppins 23035 (F) | x | x | x | x | x | - |
|  | 3907 | Greece, Spribille 18819 (GZU) | x | x | x | x | x | - |
|  | 4110 | Estonia, 55230 (TU) | x | x | x | x | x | x |
|  | 4501 | Norway, Bjerke 5-9-2010 (TLE) | x | x | - | - | x | - |
|  | MAF10225 | Spain, 10225 (MAF) | AY611092 | AY611149 | AY607804 | - | - | - |
|  | MAF10227 | Spain, 10227 (MAF) | AY611082 | AY611139 | AY607794 | - | - | - |
|  | MAF10230 | Spain, 10230 (MAF) | AY611095 | AY611153 | AY607808 | - | x | - |
| *M. exasperata*SPAIN |  |  |  |  |  |  |  |  |
|  | 6043 | Spain, 10214 (MAF) | x | x | x | x | x | x |
|  | 6044 | Spain, 7636 (MAF) | x | x | x | x | x | x |
| *M. exasperatula* |  |  |  |  |  |  |  |  |
|  | 3746 | USA, Esslinger 18855 (TLE) | x | x | x | x | x | X |
|  | 3761 | USA, Esslinger 18682 (TLE) | x | x | x | x | x | X |
|  | 3768 | USA, Henson 2/2010 (TLE) | x | x | x | x | x | X |
|  | 3775 | USA, Leavitt 09-344a (F) | x | x | x | x | x | X |
|  | 3776 | USA, Leavitt 09-344b (F) | x | x | x | x | x | X |
|  | 3779 | USA, Leavitt 09-344e (F) | x | x | x | x | x | X |
|  | 3926 | Sweden, Thell s.n. (LD) | x | x | x | x | x | X |
|  | 4116 | Estonia, 51003 (TU) | x | x | x | x | x | X |
|  | 4274 | USA, Leavitt 10.site32.01 (F) | x | x | x | x | x | X |
|  | 4277 | USA, Leavitt 10.site32.03 (F) | x | x | x | x | x | X |
|  | 4278 | USA, Leavitt 10.site32.04 (F) | x | x | x | x | x | X |
|  | 4312 | USA, Leavitt 10.site16.01 (F) | x | x | - | x | x | X |
|  | 4321 | USA, Leavitt 10.site23.01 (F) | x | x | x | x | x | X |
|  | Essl16554 | USA, Esslinger 16554 (TLE) | AY611119 | AY611175 | AY607833 | - | - | - |
|  | MAF10213 | 10213 (MAF) | AY611090 | AY611147 | AY607802 | - | **EF092124** | - |
| *M. gomukhensis* |  |  |  |  |  |  |  |  |
|  | 6049 | India, 3929 (MAF) | x | x | x | x | - | x |
|  | 6050 | India, 1109 (MAF) | x | - | - | x | x | x |
| *M. halei* |  |  |  |  |  |  |  |  |
|  | 4006 | USA, Harris 55554 (NY) | x | x | x | x | x | x |
|  | 4011 | USA, Harris 54342 (NY) | x | x | x | x | x | x |
| *M. infumata* |  |  |  |  |  |  |  |  |
|  | 3892 | USA, Spribille 52177 (KLGO) | x | x | x | x | x | x |
|  | 3960 | Greenland, ESH-08.265 (TLE) | x | x | x | x | x | x |
|  | 3961 | Greenland, ESH-08.190 (TLE) | x | x | x | x | x | x |
|  | 4888 | USA, Esslinger BP78-4 (TLE) | x | x | x | - | x | x |
| *M. laciniatula* |  |  |  |  |  |  |  |  |
|  | 3895 | Sweden, Thell s.n. (LD) | x | x | x | x | x | x |
|  | 4075 | Spain, Crespo 6027g (MAF) | x | x | x | x | x | x |
|  | 4091 | Spain, Crespo 6028h (MAF) | x | x | - | x | x | x |
|  | 4093 | Spain, Crespo s.n. (MAF) | x | x | x | x | x | x |
|  | MAF10218 | Spain, 10218 (MAF) | AY611078 | AY611135 | AY607790 | - | x | - |
|  | MAF10226 | Spain, 10226 (MAF) | AY611079 | AY611136 | AY607791 | - | - | - |
|  | MAF10231 | Spain, 10231 (MAF) | AY611094 | AY611151 | AY607806 | - | - | - |
| *M. lobulata* |  |  |  |  |  |  |  |  |
|  | 3647 | China, Wang 20084037 (SDNU) | x | x | - | x | - | - |
|  | 3661 | China, Wang 20084052-1 (SDNU) | x | - | - | - | - | - |
| *M. multispora*1 |  |  |  |  |  |  |  |  |
|  | 3762 | USA, Esslinger 18757 (TLE) | x | x | x | x | x | x |
|  | 3780 | USA, Leavitt 10-003a (F) | x | x | x | x | x | x |
|  | 4010 | USA, Lendemer 11408 (NY) | x | x | x | x | x | x |
|  | 4299 | USA, Leavitt 10.site12.01 (F) | x | x | x | x | x | x |
|  | 4301 | USA, Leavitt 10.site12.03 (F) | x | x | x | x | x | x |
|  | 4302 | USA, Leavitt 10.site12.04 (F) | x | x | x | x | x | x |
|  | Essl16555 | USA, Esslinger 16555 (TLE) | AY611123 | AY611178 | AY607837 | - | - | - |
| *M. multispora*2 |  |  |  |  |  |  |  |  |
|  | 4653 | Canada, Bjork 13320 (TLE) | x | x | x | x | - | - |
|  | 4837 | Canada, Esslinger BP100-1 (TLE) | x | x | x | x | x | x |
| *M. multispora*3 |  |  |  |  |  |  |  |  |
|  | 3750 | USA, Esslinger 18888 (TLE) | x | x | x | x | x | x |
|  | 3756 | USA, Walton 11126 (TLE) | x | x | x | x | x | x |
|  | 3763 | USA, Esslinger 18673 (TLE) | x | x | x | x | x | x |
|  | 3909 | USA, Spribille 20869 (GZU) | x | x | - | x | x | - |
|  | 3910 | USA, Spribille 20848 (GZU) | x | x | - | x | x | x |
|  | 4267 | USA, Glacy s.n. (F) | x | x | x | x | - | - |
|  | 4283 | USA, Leavitt 10.site33.02 (F) | x | - | - | - | - | - |
|  | 4344 | USA, Leavitt 10.site38.03 (F) | x | x | x | x | x | x |
|  | 4355 | USA, Leavitt 10.site40.02 (F) | x | - | - | x | x | x |
|  | 4368 | USA, Leavitt 10.site46.01 (F) | x | x | x | x | x | x |
|  | 4663 | Canada, Goward 07-292 (TLE) | x | x | - | - | x | x |
|  | 4684 | Canada, Goward 07-200a (TLE) | x | x | - | x | x | x |
|  | 4882 | USA, Esslinger BP73-4 (TLE) | x | x | x | - | x | x |
|  | 4885 | USA, Esslinger BP78-1 (TLE) | x | x | x | - | x | x |
| *M. olivacea* |  |  |  |  |  |  |  |  |
|  | 3662 | China, Ren s.n. (SDNU) | x | x | x | x | x | x |
|  | 4106 | Estonia, 45196 (TU) | x | x | - | x | - | x |
|  | 4393 | USA, McCune 30737 (OSU) | x | x | - | x | x | x |
|  | 4499 | Norway, Bjerke 6-9-2010 (TLE) | x | x | x | x | x | x |
|  | 4624 | Japan, Y. Ohmura 7132 (F) | x | x | x | x | x | x |
|  | 4665 | Norway, Goward 02-1400 (TLE) | x | x | - | - | - | - |
|  | 4869 | USA, Esslinger BP65-2 (TLE) | x | x | x | x | x | x |
|  | MAF7666 | Spain, 7666 (MAF) | x | x | x | - | x | - |
|  | UPS388154 | Sweden, 38815 (UPS) | x | - | x | - | - | - |
| *M. olivaceoides*1 |  |  |  |  |  |  |  |  |
|  | 4676 | Canada, Bjork 16460 (TLE) | x | x | x | x | - | x |
|  | 4891 | USA, Esslinger BP80-1 (TLE) | x | x | x | x | x | x |
| *M. olivaceoides*2 |  |  |  |  |  |  |  |  |
|  | 4859 | USA, Esslinger BP50-1 (TLE) | x | x | - | x | x | - |
|  | 4895 | USA, Esslinger BP81-1 (TLE) | x | x | x | x | x | - |
| *M. poeltii* |  |  |  |  |  |  |  |  |
|  | 3644 | China, Wang 20084048 (SDNU) | x | - | - | - | - | - |
|  | 3659 | China, Wang 20084093-1 (SDNU) | x | - | - | - | - | - |
|  | 6048 | India, Divakar 3929/A (MAF) | x | x | - | x | x | X |
| *M. septentrionalis* |  |  |  |  |  |  |  |  |
|  | 3682 | Scotland, Coppins 23033 (F) | x | x | - | - | - | - |
|  | 4195 | USA, Lumbsch 20113a (F) | x | x | x | x | x | X |
|  | 4197 | USA, Lumbsch 20117a (F) | x | x | x | x | x | X |
|  | 4391 | USA, McCune 30779 (OSU) | x | x | x | x | x | X |
|  | 4392 | USA, McCune 30781 (OSU) | x | x | - | x | x | X |
|  | 4862 | USA, Esslinger BP53-1 (TLE) | x | x | x | - | x | X |
|  | 4886 | USA, Esslinger BP78-2 (TLE) | x | x | - | - | - | X |
|  | Ahti60893 | Finland, Ahti 60893 (H) | AY611093 | AY611150 | AY607805 | - | - | - |
| *M. subelegantula* |  |  |  |  |  |  |  |  |
|  | 3747 | USA, Esslinger 18877 (TLE) | x | x | x | x | - | X |
|  | 3748 | USA, Walton 11095 (TLE) | x | x | x | x | - | X |
|  | 3755 | USA, Walton 11224 (TLE) | x | x | x | x | - | X |
|  | 4813 | USA, Esslinger BP117-1 (TLE) | x | x | - | - | - | X |
|  | 4887 | USA, Esslinger BP78-3 (TLE) | x | x | - | - | x | X |
|  | Essl16132 | USA, Esslinger 16132 (TLE) | AY611115 | AY611171 | AY607829 | - | x | - |
| *M. subexasperata* |  |  |  |  |  |  |  |  |
|  | 3649 | China, Wang 20084074-1 (SDNU) | x | - | - | - | - | - |
|  | 3655 | China, Du 20084031-1 (SDNU) | x | - | - | - | - | - |
| *M. subolivacea*1 |  |  |  |  |  |  |  |  |
|  | 3964 | USA, Leavitt 1036.FW5 (F) | x | x | x | x | x | X |
|  | 3979 | USA, Leavitt 1036.FW2 (F) | x | x | x | x | x | X |
|  | 3991 | USA, Leavitt 1036.FW6 (F) | x | x | x | x | x | X |
|  | 4125 | USA, Leavitt 10-317 (F) | x | x | x | x | x | X |
|  | 4127 | USA, Leavitt 10-319 (F) | x | x | - | x | x | X |
|  | 4605 | USA, Leavitt 11-015 (F) | x | x | - | x | x | X |
| *M. subolivacea*2 |  |  |  |  |  |  |  |  |
|  | 3767 | USA, Esslinger 18832 (TLE) | x | x | x | x | x | X |
|  | 3983 | USA, Leavitt 10-30.RC2 (F) | x | x | x | x | x | X |
|  | 3988 | USA, Leavitt 10-40.KC4b (F) | x | x | x | x | x | X |
|  | 4012 | USA, Lendemer 14631 (NY) | x | x | x | x | x | X |
|  | 4105 | USA, Crespo 6009k (MAF) | x | x | x | x | x | X |
|  | 4123 | USA, Leavitt 10-307 (F) | x | x | - | x | x | X |
|  | 4124 | USA, Leavitt 10-309 (F) | x | - | - | x | x | X |
|  | 4130 | USA, Leavitt 10-330 (F) | x | x | x | x | x | X |
|  | 4135 | USA, Leavitt 10-338 (F) | x | x | - | x | x | X |
|  | 4269 | USA, Kellison s.n. (F) | X | x | x | x | - | X |
|  | 4296 | USA, Leavitt 10.site10.01 (F) | X | - | x | x | x | X |
|  | 4341 | USA, Leavitt 10.site37.01 (F) | X | x | x | x | x | X |
|  | 4613 | USA, Leavitt 11-023 (F) | X | x | x | x | x | X |
|  | 4614 | USA, Leavitt 11-024 (F) | X | x | - | x | - | X |
|  | 4636 | Canada, Goward 09-89a (TLE) | X | x | x | x | x | - |
| *M. trabeculata* |  |  |  |  |  |  |  |  |
|  | 3920 | USA, Spribille s.n. (GZU) | X | x | - | x | - | X |
|  | 4857 | USA, Esslinger BP47-2 (TLE) | X | x | x | x | x | X |
|  | 4868 | USA, Esslinger BP65-1 (TLE) | X | x | x | - | x | X |
|  | 4876 | USA, Esslinger BP72-1 (TLE) | X | x | x | x | x | X |
|  | |  |  |  |  |  |  |  |
| *M. ushuaiensi*sArgentina |  |  |  |  |  |  |  |  |
|  | 6045 | Argentina, SC 1938 (MAF) | X | x | - | x | x | X |
| *M. ushuaiensi*sChile |  |  |  |  |  |  |  |  |
|  | 6046 | Chile, Chile 332 (MAF) | X | x | - | x | x | X |
|  | 6047 | Chile, Chile 337 (MAF) | X | x | - | x | x | X |
| *Melanelixia californica* |  |  |  |  |  |  |  |  |
|  | 3742 | USA, Esslinger 18915 (TLE) | X | x |  |  |  |  |
